# Innovative Blood Antioxidant Test in Standardbred Trotter Horses

**DOI:** 10.3390/antiox10122013

**Published:** 2021-12-18

**Authors:** Raffaella Rossi, Chiara Maria Lo Feudo, Enrica Zucca, Francesco Vizzarri, Carlo Corino, Francesco Ferrucci

**Affiliations:** 1 Department of Veterinary Medicine, Università degli Studi di Milano, Via Dell’Università 6, 26900 Lodi, Italy; raffaella.rossi@unimi.it (R.R.); enrica.zucca@unimi.it (E.Z.); carlo.corino@unimi.it (C.C.); francesco.ferrucci@unimi.it (F.F.); 2 Department of Agricultural and Environmental Science, University of Bari Aldo Moro, Via G. Amendola 165/A, 70126 Bari, Italy; francesco.vizzarri@uniba.it

**Keywords:** blood markers, horses, standardbreds, sports medicine, KRL test, oxidative stress, reactive oxygen species

## Abstract

In athletic horses, prolonged and intense training gives rise to an imbalance between the production of free radicals and antioxidant molecules, leading to oxidative stress. Considering the relation between exercise and oxidative stress in horses, the present work aims to validate the Kit Radicaux Libres (KRL) test as a tool to verify the influence of taming, training and racing on the total blood antioxidant activity and some haematochemical parameters. Five Italian Standardbred racehorses (two males and three females, aged 12 ± 1 months) from the same training center were selected and monitored upon arrival and during the following year until the racing season. Blood samples were obtained at different timepoints, corresponding to different steps of training. The data showed that KRL values were higher (*p* < 0.001) before the beginning of the taming period and at 60 days of taming, compared with the training and racing periods; additionally, the total protein value was affected by the training program, whereas no effects of training on muscle enzymes were detected. These results confirm that exercise plays a role in the production of free radicals and show that the KRL test may represent a valid method to determine oxidative stress in athletic horses.

## 1. Introduction

In a living being, a correct balance between oxidants and antioxidants is essential for physiological functions; in fact, reactive oxygen species (ROS) are continuously produced by the organism through several biochemical processes [1]. If the generation of ROS exceeds the capabilities of the antioxidant systems to neutralize them, oxidative stress condition is established and cell damages occur, leading to apoptosis [2].

During its evolution as an animal grazer, the horse developed particular features such as speed and resistance, becoming a great athlete. In trotter horses, prolonged and intense training activity determines an imbalance between the production of free radicals and antioxidant molecules, leading to oxidative stress. Moreover, exercise has proved to be positively correlated with increasing activities of erythrocytes superoxide dismutase [3]. The degree of stress oxidation and the values of muscle enzymes depend on the age of the horse, its level of training, and the intensity of the exercise to which it is subjected. During intensive exercise program, erythrocytes become more vulnerable to oxidative damage depending on the performance of active oxygen forms and high concentrations of polyunsaturated fatty acids and haemoferrum [4,5]. Horses training performance is accompanied by the development of tissue hypoxia; therefore, it is important to study in detail the changes of the hematopoiesis indices responsible for providing oxygen to tissues.

Nevertheless, the health and welfare of the athletic horses may be improved by completing their diet with antioxidant substances before performing intense workouts [6]. When the level of training improves, the workout ameliorates the organism’s defensive capabilities, although prolonged periods of training and races may impact the antioxidant/oxidant balance [7]. Supplementation of antioxidants such as vitamin E, vitamin C, and lipoic acid may be beneficial for equine athletes by increasing the antioxidant capacity, thus decreasing oxidative stress markers and muscle enzymes [8].

On this basis, it is extremely important to know the functional and metabolic processes occurring during exercise, for understanding which metabolic paths are involved, and which physiological processes are induced during different types of physical activity [9]. When monitoring animal health and welfare, hematological and biochemical tests can detect a disease in the early stages, since blood is a sensitive indicator of metabolic disorders, and both physiological and pathological conditions [10]. The Kit Radicaux Libres (KRL) test is a biological test which allows to determine the total blood antioxidant activity under controlled and standardized conditions. Several in vitro and in vivo applications of this test have been reported, such as the evaluation of the antioxidant activities of natural extracts [11] and of the animal antioxidant status in relation to dietary antioxidants and housing condition [12,13].

Since exercise has been related to oxidative stress in sport horses, the present study aims to validate the KRL test as a tool to evaluate the possible influence of intense exercise (training and racing) on the total blood antioxidant activity in Standardbred trotter horses. Moreover, in the same subjects, the hematological and biochemical parameters during taming, training, and racing will be evaluated.

## 2. Materials and Methods

### 2.1. Animals and Diet

All the described animal-related procedures were conducted according to Directive 2010/63/EU of the European Parliament and of the Council of 22 September 2010 on the protection of animals used for scientific purposes (Article 1, Paragraph 1, Letter b) and the Italian legislation (D. Lgs. n. 26/2014, Article 2, Paragraph 1, Letter b). 

Five Italian trotter horses (two males and three females) homogeneous for age (12 ± 1 months) were selected. All horses came from the same breeding farm and were subjected to the same feeding regimen and housing. Horses were individually fed 3 meals/day (h 7.00, 13.00, 19.00) to cover the intake requirements. The diet of each horse was composed of 8 kg/day of mixed alfalfa hay, 7 kg/day of cereals (barley 70%, oats 20%, corn 10%), and a dietary supplementation (protein 15%, fiber 5.5% and fat 5%) to meet the requirements [14]. The horses were offered water ad libitum.

### 2.2. Training Program

The horses were monitored upon their arrival at the training centre until the racing season of the following year. The period of taming began 15 days after the arrival. After four months of taming, the training phase began in preparation to the races, starting one year after the arrival. The training phase included different training sessions. Three times per week, an aerobic training at a moderate speed, called “train”, was performed. Every 3–4 days, an intensive workout was planned, identified as “test”; tests were based on the “interval training” technique, consisting of carrying out repeated runs, alternated with recovery at walk. The day after the test, horses were kept on active rest in the paddock. Table 1 shows an exemplary weekly training program of the enrolled horses.

### 2.3. Blood Collection

All horses underwent blood collection throughout the whole experiment. Blood samples were collected after 12 h of fasting from the jugular vein using 10 mL vacutainer glass tubes (Venoject**^®^**, Terumo Europe N.V., Leuven, Belgium) containing Ethylenediaminetetraacetic acid (EDTA) and immediately stored at 4 °C. The samplings were carried out at different timepoints: 15 days after the arrival at the training centre, at the start of the taming period (T0), at 60 days from the start of the taming period (T1), at 120 days, at the beginning of the training period (T2), at 180 days, two months after the start of the training period (T3), at 270 days, four months after the start of training period (T4), at 330 days, six months after the start of the training period (T5), at 390 days, eight months after the start of the training period and at the beginning of the racing season (T6), and at 450 days, two months after the start of the racing season (T7). All the samplings were performed 12 h after the end of the daily exercise to avoid its effect on hematological and biochemical parameters.

### 2.4. Hematological and Biochemical Parameters

The hematological and biochemical parameters were assessed within 12 h from sample collection. The following parameters were determined using a hematological cell counter (Cell Dyn 3500 Plus, Abbott Diagnostics Division, Mountain View, CA, USA): erythrocytes (RBC), hemoglobin (Hgb), hematocrit (Hct), mean corpuscular volume (MCV), mean corpuscular hemoglobin (MCH), mean corpuscular hemoglobin concentration (MCHC), leukocytes (WBC), neutrophils, lymphocytes, monocytes, eosinophils and basophils. Biochemical parameters were assessed on plasma samples, using an automated biochemistry analyzer (Cobas Mira Classic, Roche, Basel, Switzerland), and included: total proteins (PT), creatine phosphokinase (CPK) and aspartate aminotransferase (AST).

### 2.5. Blood Antiradicalic Activity

The principle of the KRL test is to submit whole blood to thermo-controlled free radical aggression to mobilize all families of any free radical scavengers in the blood to neutralize the oxidation processes [15,16,17]. All the chemical and enzymatic antioxidant systems of the sample were triggered to protect cells integrity until lysis. The total antiradical activity of whole blood and RBC for each horse at the different timepoints was evaluated using the KRL biological test (Laboratoires Spiral, Couternon, France). Whole blood and RBC samples were submitted in an isotonic saline solution to organic free radicals produced at 37 °C from the thermal decomposition of a solution of 2.20-azobis (2-amidinopropane) dihydrochloride (AAPH) (Kirial International, Dijon, France). Haemolysis was recorded using a 96-well microplate reader by measuring the optical density decay at 450 nm. For each well, absorbance measurements were performed 75 times, once every 150 s. Results were expressed as the time required to reach 50% of maximal haemolysis (KRL value, in min), which refers to the whole blood resistance to free-radical attack. Intra and inter-assay coefficients of variation of the KRL test were 2.5% and 4%, respectively.

### 2.6. Statistical Analysis

The data concerning haematochemical parameters and KRL value were analyzed using a commercially available statistical software package (SPSS/PC Statistics 24 SPSS Inc., IBM, Armonk, NY, USA). A univariate analysis of variance (ANOVA) was performed with sampling time as an effect to highlight the influence of training on the considered parameters. The means were compared by means of the Student–Neuman–Keuls test. Data are presented as means ± SEM, and statistical significance was set at *p* < 0.05.

## 3. Results

### 3.1. Antiradicalic Activity

**Figure 1:**
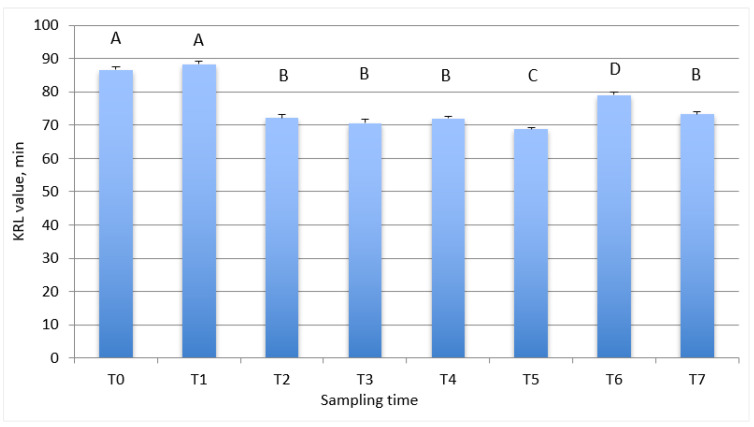
Total antiradicalic activity of whole blood in relation to the training program of the trotter horses. Data are reported as mean ± SEM. T0, start of taming period; T1, 60 days from start of taming period; T2, beginning of training period; T3, 60 days after the start of training period; T4, 120 days after the start of training period; T5, 180 days after the start of training period; T6, beginning of racing season; T7, 60 days after the beginning of racing season. A, B, C, D differs for *p* < 0.001.

The KRL values in whole blood in relation to the training program of the trotter horses are reported in Figure 1; in particular, the figure shows that horses at the start of the taming period and after 60 days of taming (T0 and T1) have a higher (*p* < 0.001) KRL value compared with the other sampling times. At the beginning of the training period and during the subsequent four months (from T2 to T4), a significant decrease in KRL value (*p* < 0.001) was observed. At the end of training (T5) a lower value was also reported. An increase (*p* < 0.001) of this value was observed at the beginning of the racing season (T6), even if the values remain lower than those observed at the beginning of the study (T0 and T1 sampling). At the last sampling time, corresponding to two months after the start of the racing season (T7), the KRL values were comparable with those observed during the training phases (from T2 to T4).

**Figure 2:**
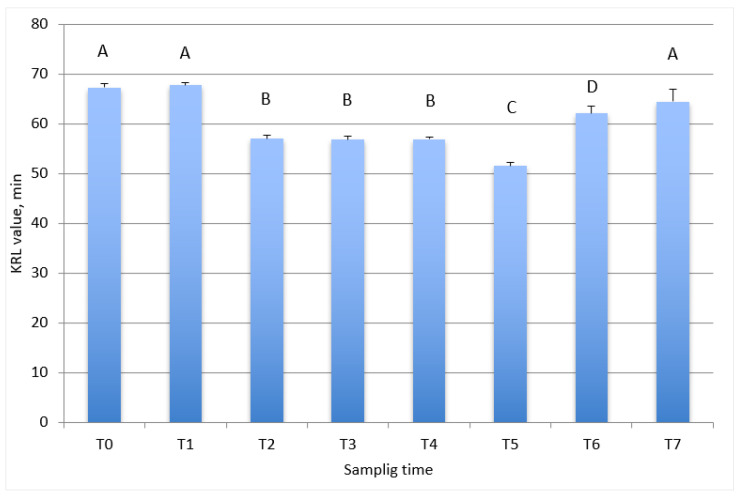
Total antiradicalic activity of red blood cells in relation to the training program of the trotter horses. Data are reported as mean ± SEM. T0, start of taming period; T1, 60 days from start of taming period; T2, beginning of training period; T3, 60 days after the start of training period; T4, 120 days after the start of training period; T5,180 days after the start of training period; T6, beginning of racing season; T7, 60 days after the beginning of racing season. A, B, C, D differs for *p* < 0.001.

In Figure 2, the total antiradicalic activity of red blood cells in relation to the training program of the trotter horses is reported. At the first two sampling times (T0 and T1), corresponding to beginning of timing and 60 days after the beginning of taming, higher KRL values were observed compared with training/racing samplings. At the beginning of the training period and during the subsequent four months (from T2 to T4) a significant decrease in KRL value (*p* < 0.001) was observed. At six months after the start of the training period, the lowest value of KRL was observed (T5). At the beginning of the racing season (T6), the KRL value of red blood cells increased (*p* > 0.001) compared with the previous value and, after 60 days (T7), the KRL values were comparable with those observed during the taming period (T0 and T1).

### 3.2. Haematological and Biochemical Parameters

In Table 2, the haematological parameters of trotter horses in relation to the training program are reported. RBC, WBC, Hgb, and Ht were unaffected (*p* > 0.05) by training program and fell within the reference values. MCV, MCH, and MCHC varied (*p* < 0.001) in relation to the training program. These parameters increased during the training and reached the highest values during the racing season. Additionally, the leukocyte formula was affected (*p* < 0.001) by training program. Neutrophil percentage was higher in the first two sampling (T0 and T1), decreased during training (from T2 to T6), reaching the lowest value at 60 days after the beginning of the racing season. Lymphocytes presented an opposite trend, with the highest value at the last sampling time (T7). Monocytes, eosinophils and basophils did not present a linear trend during training and racing.

The haematochemical parameters (PT, CPK, and AST) in relation to the training program are reported in Table 3. The PT values are affected by the training program, with the lowest value at the least sampling time (T7). Instead, the statistical analysis did not highlight any significant difference for CK and AST at the different sampling times (AST, *p* = 0.636 and CPK, *p* = 0.204), and all the values fell within the reference values for these biochemical parameters in athletic horses.

## 4. Discussion

In the literature, evidence that oxidative stress occurs in horses during exercise has been reported [18,19], related to work intensity, animals’ age, and training levels [20]. The production of ROS molecules is fundamental for training adaptation and to boost the muscle force [21]; however, if ROS are in overflow, they may damage cellular components, increasing the risk of the development of pathologic conditions that may impair the performance [22]. 

The present study is the first one which evaluates the antioxidant status in Italian Standardbred trotter horses using the biological test KRL. The KRL value reflects the organism’s antioxidant status and provides useful information about a subject’s capability to counteract several stressors, denoting the resistance of the organism to the attack of free radicals. The obtained data evidence how the antioxidant status of the enrolled horses significantly varied in relation to the different phases: taming, training, and racing. As shown in Figure 1 and Figure 2 during the first few months of experimental period the horses showed a high KRL value in blood and RBC, which reflects a good antioxidant status. This finding suggests that the horses came from ideal breeding condition: in fact, before being transferred to the training center, they lived in a stable group of horses in a paddock. At the third sampling, corresponding to the beginning of training, the situation changed, as the intensification of the physical effort represented a stressful event for the horses. This situation led to a significant decrease in the KRL value, which remained stable over time of training. This finding is in agreement with the data reported by White et al. (2001) [19], showing that, in horses, exercise plays a role in the production of free radicals, thus leading to oxidative stress status. The KRL value remaining constant during the training phase underlines that the trainer managed the animals in a proper way. 

An increase in the KRL value at the beginning of the racing season compared with the training period was observed; this result may be related to a period of lower intensity training just before the start of the racing season, aimed to guarantee horses to reach competition level in an optimal situation.

At the last sampling time, two months after the start of the racing season, the KRL values decreased and were comparable with the values observed during the training. 

The present study shows that the KRL test reveals a high sensitivity also in trotter horses to monitor the antioxidant status in relation to training phases. Previous studies using the KRL test underline its high sensitivity in detecting the effects of dietary antioxidants and different housing condition in pigs [11,12]. Considering the data obtained by the current study on antioxidant status during training and racing and those reported by the scientific literature, dietary integration with antioxidant such as vitamin E and selenium, should be considered in sport horses in order to reduce exercise-induced oxidative stress [23]. Different dietary integrations such as natural extracts, and their respective dosages could be tested in relation to horse’s age and type of training [24].

In the present study, no significant change of CPK and AST levels was detected. The increase in the plasma concentrations of enzymes/molecules present within skeletal muscle cells such as CPK and AST may be expected due to muscle damage or intense muscle effort [25]. In particular, increased AST during training has been related to a loss or a modification in the membrane of the muscle fiber, determining a temporarily increased permeability [26].

The present data underline that there is an adaptation in relation to training and racing and that animals were managed properly by the trainer; in fact, the time young horses needed for adapting to a new training level was respected, without exceeding with extreme workouts, which could have been excessive for their muscle-skeletal condition.

## 5. Conclusions

The results of the present study highlight that the KRL biological test has demonstrated a high sensitivity in assessing blood antioxidant status in young Standardbred racehorses and has proved to represent a valid method to determine oxidative stress in the athletic horse. Therefore, this analytical tool may find practical applications for the monitoring of the antioxidant status in trotter racehorses. Further investigations are needed in order to evaluate antioxidant status with the KRL test in other sport disciplines and to select the most appropriate dietary antioxidants supplementation to protect athletic horses from oxidative stress.

## Figures and Tables

**Table 1:** Example of a weekly training program of the horses.

Monday	Tuesday	Wednesday	Thursday	Friday	Saturday	Sunday
Test	Paddock	Train	Train	Test	Paddock	Train

**Table 2:** Haematological parameters of trotter horses in relation to the training program.

Item	Sampling Time								*p* Value
	T0	T1	T2	T3	T4	T5	T6	T7	
RBC, ×10^3^/mm	10,526 ± 571.3	9953 ± 564.2	982 ± 389.9	9362 ± 632.8	9581 ± 329.3	10,303 ± 796.3	9976 ± 413.4	8475 ± 363	0.286
WBC, mmc	8288 ± 406.3	7886 ± 548	8685 ± 682.2	8567 ± 370.9	10,508 ± 1153.2	7675 ± 386.9	9374 ± 1313	9066 ± 873	0.212
Hgb, g/dL	14.3 ± 0. 8	13.8 ± 0.8	13.7 ± 0.5	13.1 ± 0.8	12.1 ± 0.4	14.8 ± 1.13	14.9 ± 0.6	12.9 ± 0.3	0.179
Htc, %	42.3 ± 2.1	40.4 ± 1.9	40.8 ± 1.4	39.2 ± 2.5	40.7 ± 1.5	43.5 ± 2.9	43.2 ± 1.6	37.2 ± 1.1	0.465
MCV, μ^3^	40.2 ± 0.5	40.7 ± 0.6	41.5 ± 0.5	42.3 ± 0.6	42.5 ± 0.4	42.4 ± 0.6	43.3± 0.6	44.7 ± 0.5	<0.001 ***
MCH, μμ g	13.6 ± 0.1	13.8 ± 0.1	14 ± 0.2	13.9 ± 0.1	12.6 ± 0.1	14.3 ± 0.2	14.9 ± 0.2	15.7 ± 0.2	<0.001 ***
MCHC, g/dl	33.7 ± 0.3	34.1 ± 0.4	33.6 ± 0.2	33.3 ± 0.2	29.8 ± 0.1	33.9 ± 0.3	34.4 ± 0.1	35.1 ± 0.1	<0.001 ***
Neutrophil, %	54.1 ± 1.5	58.5 ± 2.0	49.6 ± 1.7	53.7 ± 3.5	61.3 ± 7.5	55 ± 3.9	46 ± 4.8	38.2 ± 2.7	0.010 *
Lymphocyte %	39.1 ± 1.4	36 ± 2.0	41.4 ± 0.5	43.5 ± 0.9	32 ± 5.6	42.3 ± 3.7	51.6 ± 4.9	59.4 ± 2.4	<0.001 ***
Monocyte, %	4.1 ± 0.6	0.20 ± 0.1	1.67 ± 0.5	1.82 ± 0.4	2.0 ± 0.5	2.3 ± 0.3	1.6 ± 0.7	1.6 ± 0.8	0.009 **
Eosinophil, %	2.5 ± 0.5	5.3 ± 0.7	6.1 ± 0.8	4.2 ± 0.9	4.5 ± 1.2	0.3 ± 0.01	0.8 ± 0.1	0.80 ± 0.1	<0.001 ***
Basophil, %	0	0	0	0.01 ± 0.007	0.17 ± 0.167	0	0	0.2 ± 0.02	0.576

The data are reported as mean ± SEM. RBC, red blood cells; WBC white blood cells, Hgb, hemoglobin, Htc, hematocrit, mean corpuscular volume, mean corpuscular volume, MCH Mean corpuscular hemoglobin, MCHC, mean corpuscular hemoglobin concentration, T0, start of taming period; T1, 60 days from start of taming period; T2, beginning of training period; T3, 60 days after the start of training period; T4, 120 days after the start of training period; T5, 180 days after the start of training period; T6, beginning of racing season; T7, 60 days after the beginning of racing season. The statistical significance is shown as * (*p* < 0.05), ** (*p* < 0.01), *** (*p* < 0.001).

**Table 3:** Biochemical parameters of trotter horses in relation to the training program.

Item	Sampling Time								*p* Value
	T0	T1	T2	T3	T4	T5	T6	T7	
PT g/dL	5.4 ± 0.2	5.9 ± 0.1	5.6 ± 0.04	5.6 ± 0.09	5.8 ± 0.05	6.0 ± 0.09	5.8 ± 0.08	5.2 ± 0.05	<0.001 ***
AST, U/L	403.3 ± 11.1	373.6 ± 52.8	644.1 ± 126.5	464.1 ± 150.6	461.6 ± 112.7	267.1 ± 46.1	311.8 ± 51.8	414 ± 91.5	0.636
CPK, U/L	281.5 ± 49,6	216.5 ± 16	321.8 ± 35.3	213.3 ± 23.6	873.1 ± 229	158.8 ± 15.2	1092.2 ± 339.4	178 ± 17.5	0.204

The data are reported as mean ± SEM. PT, total protein; CPK, creatine phosphokinase; AST, aspartate aminotransferase;T0, start of taming period; T1, 60 days from start of taming period; T2, beginning of training period; T3, 60 days after the start of training period; T4, 120 days after the start of training period; T5, 180 days after the start of training period; T6, beginning of racing season; T7, 60 days after the beginning of racing season. The statistical significance is shown as *** (*p* < 0.001).

## Data Availability

The data is contained within the article.
